# The Impact of a Novel Mimicry Task for Increasing Emotion Recognition in Adults with Autism Spectrum Disorder and Alexithymia: Protocol for a Randomized Controlled Trial

**DOI:** 10.2196/24543

**Published:** 2021-06-17

**Authors:** Joshua A Caine, Britt Klein, Stephen L Edwards

**Affiliations:** 1 School of Science Psychology and Sport Federation University Australia Ballarat Australia; 2 Deputy Vice Chancellor of Research & Innovation Portfolio Federation University Australia Ballarat Australia; 3 Health Innovation and Transformation Centre Federation University Australia Ballarat Australia; 4 Biopsychosocial and eHealth Research & Innovation Federation University Australia Ballarat Australia

**Keywords:** alexithymia hypothesis, training facial expression emotion recognition, mimicry task, autism spectrum disorder, interoception, facial expression, emotion, emotion recognition, autism, spectrum disorder, mimicry, therapy, protocol, expression, disability

## Abstract

**Background:**

Impaired facial emotion expression recognition (FEER) has typically been considered a correlate of autism spectrum disorder (ASD). Now, the alexithymia hypothesis is suggesting that this emotion processing problem is instead related to alexithymia, which frequently co-occurs with ASD. By combining predictive coding theories of ASD and simulation theories of emotion recognition, it is suggested that facial mimicry may improve the training of FEER in ASD and alexithymia.

**Objective:**

This study aims to evaluate a novel mimicry task to improve FEER in adults with and without ASD and alexithymia. Additionally, this study will aim to determine the contributions of alexithymia and ASD to FEER ability and assess which of these 2 populations benefit from this training task.

**Methods:**

Recruitment will primarily take place through an ASD community group with emphasis put on snowball recruiting. Included will be 64 consenting adults equally divided between participants without an ASD and participants with an ASD. Participants will be screened online using the Kessler Psychological Distress Scale (K-10; cut-off score of 22), Autism Spectrum Quotient (AQ-10), and Toronto Alexithymia Scale (TAS-20) followed by a clinical interview with a provisional psychologist at the Federation University psychology clinic. The clinical interview will include assessment of ability, anxiety, and depression as well as discussion of past ASD diagnosis and confirmatory administration of the Autism Mental Status Exam (AMSE). Following the clinical interview, the participant will complete the Bermond-Vorst Alexithymia Questionnaire (BVAQ) and then undertake a baseline assessment of FEER. Consenting participants will then be assigned using a permuted blocked randomization method into either the control task condition or the mimicry task condition. A brief measure of satisfaction of the task and a debriefing session will conclude the study.

**Results:**

The study has Federation University Human Research Ethics Committee approval and is registered with the Australian New Zealand Clinical Trials. Participant recruitment is predicted to begin in the third quarter of 2021.

**Conclusions:**

This study will be the first to evaluate the use of a novel facial mimicry task condition to increase FEER in adults with ASD and alexithymia. If efficacious, this task could prove useful as a cost-effective adjunct intervention that could be used at home and thus remove barriers to entry. This study will also explore the unique effectiveness of this task in people without an ASD, with an ASD, and with alexithymia.

**Trial Registration:**

Australian New Zealand Clinical Trial Registry ACTRN12619000705189p; https://www.anzctr.org.au/Trial/Registration/TrialReview.aspx?id=377455

**International Registered Report Identifier (IRRID):**

PRR1-10.2196/24543

## Introduction

### Background

Behavioral and cognitive neuroscience has elaborated a range of emotion-related difficulties with emotion recognition [1], empathy [2], and regulation of emotions in autism spectrum disorder (ASD) [3]. However, these abnormalities are not observed in all people with ASD [4]. Traditionally, these emotion-processing difficulties have been viewed as inevitable correlates of ASD, but recent research suggests that their presence is variable and better explained by co-occurring alexithymia. This suggestion is termed the alexithymia hypothesis [5] and is gaining significant support [6]. Alexithymia is a personality construct that relates to the inability to distinguish or describe the subjective feelings in the body produced by emotion. Alexithymia is related to both empathy [7] and emotion recognition [8] deficits. Severe degrees of alexithymia are manifested in up to 65% of adults with ASD [9].

The 2 forms of alexithymia, type 1 and type 2 [10], differ on the affective and cognitive dimensions [11]. Type 1 alexithymia includes high levels of both dimensions, whereas type 2 alexithymia only includes high levels of the cognitive dimension. Type 1 relates to reduced emotional reactivity (or a severe downregulation of emotional experience), and type 2 is typical emotional reactivity, but a reduced ability to interpret and describe that emotion [12].

### Simulation Theories

Simulation theories [13] may explain how these emotion processing deficits occur in alexithymia. Simulation theories refer to the perception of a facial expression triggering a simulation of that expression's corresponding emotion within the perceiver. This simulation is then used to understand the perceived facial expression meaning to facilitate emotion recognition and empathy [14,15].

Alexithymia can explain the failure of this simulation process. Studies suggest that reduced emotion recognition accuracy in alexithymia is related to reduced activity in the insula (a small region within the cerebral cortex) [16]. The insula is related to the cognitive processing of emotions and generation of emotions [17,18], which are both required for simulation to work. The insula has also been noted as a critical relay station between the action representation system, which represents the vision of facial expressions, and limbic areas, which generate emotion [19]. In alexithymia, it has been found that the insula is hypoactive [12]. Therefore, when a facial expression is seen, the insula may fail to relay this information to the limbic area, which typically simulates the observed emotion.

The simulation process may also fail in alexithymia due to an inability to access the emotional information generated by a simulation. There are 2 explanations as to why people with alexithymia may not be able to access the simulation information. The first explanation is because of the inability to describe emotions in the self and differentiate them from bodily sensations, which is the hallmark of alexithymia [20]. Second, significant interoceptive deficits have been observed in alexithymia, which explains why people with alexithymia cannot accurately assess their current emotional state [21,22]. This interoceptive deficit would suggest that, even if an emotion was generated during simulation, people with alexithymia would not be able to appraise and understand this simulated emotion. Therefore, the simulation process would not provide any information about what the other person is feeling.

Another way that alexithymia may explain a failure of the simulation process is if people with alexithymia have trouble assigning the simulated emotion as belonging to the “sender” of the emotional expression [23], which is termed self-other distinction. If an emotion is simulated, but not tagged as belonging to the “sender,” then the simulation cannot aid recognition of the sender's emotion.

While alexithymia is a key element in explaining the failure of this simulation process, it does not provide a finer mechanistic explanation. However, theories proposed within predictive coding frameworks (PCFs) are beginning to provide a compelling explanation.

### Predictive Coding Frameworks, Interoception, and Self-Other Distinction

PCFs suggest that the brain uses complex mathematical models to predict the most likely truths occurring in ourselves and our worlds [24,25]. These models are continually updating to increase efficiency and accuracy at explaining the most probable cause of sensory inputs [24]. Quattrocki and Friston [26] proposed a PCF theory that suggests that the causal factor of ASD is an aberrant oxytocin system. They proposed that the neuropeptide oxytocin has 2 functions. First, it has a role in prescribing the perceived accuracy of an interoceptive signal within the body. The perceived accuracy of an interoceptive signal is important because, if a top-down prediction and bottom-up stimulus do not match up, the brain will rely more heavily on the information source it perceives to be most accurate.

Therefore, when generating a model of the current state of the self or body, oxytocin mediates how much weight will be given to an interoceptive stimulus versus predictive models of the body’s current state. Second, oxytocin has a role in enabling the plasticity required for the brain to generate a model of the emotional and social “self.” Without these models, an over reliance would be put on bottom-up information, as described by Pellicano and Burr [27].

Two constructs implicated in forming a distinction between the self and others were introduced in the earlier paragraphs: interoception and the self. Interoception is the perception of one’s own internal state, including the perception of sensations such as thirst, stress, temperature, sleepiness, and heartbeat. One use of these sensations is for inferring emotional states and generating a model of the embodied “self” [25]. The embodied “self” is the physical body belonging to an individual, which the brain distinguishes as separate from other matter in the environment [28]. These bottom-up stimuli inform limbic brain regions and are suggested to assimilate with higher-order models of the self in the insula, anterior cingulate cortex, and ventromedial prefrontal cortices [29,30]. Therefore, these 3 areas are responsible for producing top-down predictions of perceptual information that has an emotional valence (ie, “gut feelings”). These emotionally valenced predictions are key components of the subjective emotional experience and self-awareness [25]. Quattrocki and Friston [26] suggested that one way in which models of the self can be weak is due to an aberrant oxytocin system. They suggest that an aberrant oxytocin system results in a failure to attribute less accuracy to interoceptive cues, which leads to a biased use of interoceptive cues over top-down prediction models. This biased use of interoceptive cues results in the models of the self being underdeveloped, lacking efficiency and accuracy. Underdeveloped models of the self make the ability to form a self-other distinction exceedingly difficult, and therefore, the ability to attribute an emotion to another person is compromised. Consequently, any emotional presentation generated via simulation for either empathy or emotion recognition may be experienced, however, not able to be attributed to the other person.

### Facial Mimicry and Oxytocin

Facial mimicry and oxytocin may aid these interoceptive and emotional processing symptoms. Aoki and colleagues [31] showed that activity in the insula could be increased during emotion recognition tasks in people with ASD. They found that oxytocin significantly increased emotion recognition (*P*=.043) and increased activity in the insula during these tasks (*P*=.004). This study did not measure alexithymia; however, emotion recognition deficits in ASD have been found to become insignificant after accounting for alexithymia [32]. Therefore, this relationship between emotion recognition, oxytocin, and activity in the insula may actually be more applicable to alexithymia than to ASD.

One natural way to increase oxytocin has been suggested by Delaveau et al [33] by using mimicry. Mimicry referrers to copying the movements of another person. Delaveau et al [33] found that when mimicking and being mimicked, there was increased activity in the insula, similar to the results of Aoki and colleagues [31]. Paired with prior behavioral studies [34], the authors suggested that these results may be due to mimicry increasing the salience of social cues through the modulation of brain regions related to self-other processing. Further, they suggested that the systematic use of mimicry may provide therapeutic benefits. Given that both oxytocin and self-other processing have been implicated as key targets for the emotional processing problems observed in alexithymia, an intervention that includes mimicry may be a novel and simple therapeutic method.

Lewis and Dunn [35] offered support for the effectiveness of instructed mimicry in their emotion recognition study. They found that instructions to mimic a facial expression improved the recognition of that emotional expression selectively for people with high autistic traits. This finding was similar to that of Luminet et al [36] who found that oxytocin selectively benefits people high in alexithymia on a similar task. Additionally, other studies have shown that by restricting facial mimicry, facial emotion expression recognition (FEER) decreases (eg, [37-39]).

### Study Aims

The first aim of this study is to evaluate the efficacy of an instructed mimicry task condition to improve FEER compared to a control condition and also from baseline to posttest in adults with and without alexithymia and ASD. The second aim of the study is to confirm findings by Cook et al [32] that FEER is related to alexithymia and not ASD. As an ancillary aim, the study will also collect data for further research on how eye-gaze patterns in type 1 alexithymia and type 2 alexithymia uniquely relate to FEER in the same sample.

## Methods

### Study Design

The study design is a mixed factor 2x2x2 within-between design. Participants who meet inclusion and exclusion criteria will be randomized to a control task or the experimental task. Inclusion and exclusion criteria and pretrial control variables will be collected during an online screening stage and during an in-person clinical interview. Baseline outcomes will be assessed directly before the control task or experimental task. They will then be assessed again during the control and experimental tasks. The study will conclude with a brief measure of satisfaction of the task and a debriefing session. See Multimedia Appendix 1 for a step-by-step workflow of a participant through the study.

### Participants

We will recruit through local ASD support groups, online social media, and noticeboard advertisements (eg, at Federation University Australia). We will also use snowball recruiting to better penetrate the ASD community. Participants with an ASD (based on the assessments described in the Assessment section) will be assigned to the ASD group. Other participants will be assigned to the alexithymia-matched general population group (AM group).

The researchers will also use the 20-item Toronto Alexithymia Scale (TAS-20) total scores [40] to match the ASD group with the AM group without an ASD. Specifically, the number of participants in each group meeting criteria for alexithymia will be matched as indicated by TAS-20 scores ≥61, which is how groups have been matched in similar studies (eg, [32]).

The inclusion criterion is that participants are 18 years of age and older. The ASD group also has the added inclusion criterion of a confirmed ASD based on the assessment described in the Assessment section. Exclusion criteria include the participant having another mental health condition (other than an ASD if in the ASD group) diagnosed by a clinician and that the Kessler Psychological Distress Scale (K10) [41] indicates a significant level of distress (cut-off score of 22).

### Assessments

The main aim of the current study is to assess the efficacy of the intervention in the general population and an ASD population, but also if people with high levels of alexithymia gain additional benefit. Further, this study will aim to replicate the study by Cook et al [32], which suggested that alexithymia, not ASD, predicts poor FEER. This requires having a general population group and another group with people with ASD, with both groups being matched on levels of alexithymia. Therefore, participants will be prescreened online to facilitate creating this composition of participants. During the online screening stage, participants will choose to consent in the study, complete the TAS-20, and indicate if they have an ASD or if they have been diagnosed with an ASD. During this screening stage, participants will also complete demographic information, the Autism Spectrum Quotient (AQ-10) [42], and the K-10.

Participants will then be invited to a clinical interview with a provisional psychologist (PP) at the Federation University Psychology Clinic who will confirm a reported diagnosis of ASD and complete an observational assessment that gives a diagnostic indication of an ASD. Participants who have reported a diagnosis will be asked what instrument was used and if the PP can request a copy of the results. The PP will also complete the Autism Mental Status Exam 2.2 (AMSE) [43,44]. The AMSE is an 8-item observational assessment that takes place during a clinical interview and predicts ASD classification on the Autism Diagnostic Observation Schedule (ADOS) [45]. During this clinical interview, participants will also be administered the Depression Anxiety Stress Scales-21 (DASS-21) [46] and the Wechsler Abbreviated Scale of Intelligence (WASI-II) [47] to assess depression, anxiety, and IQ, which will be used as control variables in the analyses of the results.

### Assessor Training and Supervision

The PPs will have pre-existing training in conducting clinical interviews, including mental status examination, as well as administration of symptom measures and cognitive testing. They will be supervised by approved supervisors and endorsed clinical psychologists with extensive clinical experience (at least 10 years) working with and diagnosing people who have an ASD. All psychologists will participate in the AMSE training [48], which entails working through the AMSE manual, examples, and posttests. The viewing, rating, and discussion of additional videos of adults with ASD will enhance interrater reliability and ensure understanding of diagnostic criteria.

Clinical interviews will be recorded, and AMSE scoring decisions will be made with one or, where it is difficult to reach consensus, both of the supervisors. Participants who meet AMSE criteria and have a confirmed historical diagnosis will be included in the ASD group.

### Measures

During the screening stage of the study, participants complete the K-10, TAS-20, AQ-10, and several single-item questions pertaining to demographic variables and known presence of other mental conditions. In the clinical interview with the PP, participants will be administered the DASS-21 and WASI-II, and the clinician will also use this time to complete the AMSE observational measure. Indication of context and time-point for each measure can be seen in Table 1.

**Table 1:** Study design and measures.

Concept	Measure	Instrument	Condition/ time point				
			Online screening stage	Clinical interview stage	Experiment stage		
					Pretest	During Experimental task	Posttest
Primary outcome	FEER^a^ ability	FEER task Improvement score	-	-	X	X	-
Secondary Outcome	Acceptance / Enjoyment of task	Task satisfaction Questionnaire	-	-	-	-	X
Eligibility	Psychological distress	K-10^b^	X	-	-	-	-
	Depression and Anxiety	DASS-21^c^	-	X	-	-	-
	Predict ASD^d^ classification on the ADOS^e^	AMSE^f^	-	X	-	-	-
	Predict ASD classification	AQ-10^g^	X	-	-	-	-
	Alexithymia	TAS-20^h^	X	-	-	-	-
Control variables	Alexithymia	BVAQ^i^	-	-	X	-	-
	IQ	WASI-2^j^	-	X	-	-	-
	Demographics	Demographic information	X	-	-	-	-

^a^FEER: facial emotion expression recognition.

^b^K-10: Kessler Psychological Distress Scale.

^c^DASS-21: Depression Anxiety Stress Scales-21.

^d^ASD: autism spectrum disorder.

^e^ADOS: Autism Diagnostic Observation Schedule.

^f^AMSE: Autism Mental Status Exam.

^g^AQ-10: Autism Spectrum Quotient.

^h^TAS: Toronto Alexithymia Scale.

^i^BVAQ: Bermond–Vorst Alexithymia Questionnaire.

^j^WASI-2: Wechsler Abbreviated Scale of Intelligence.

#### K-10

The K-10 [41] is a measure of psychological distress. It consists of 10 items measured using 5 values that ask how often a specific feeling was felt over the past 4 weeks. Possible answers range from 1 “None of the time” to 5 “All of the time,” with higher values indicating more distress. Possible scores range from 10, indicating no distress, to 50, indicating severe distress. A cut-off score of 19 will be used to indicate the presence of case-level anxiety or depression [49]. The K-10 is a reliable and valid measure of psychological distress with high discriminant validity [41].

#### TAS-20

The 20-item TAS-20 [40] is the most widely used measure of alexithymia. It consists of 20 items, with each item being rated on a 5-point Likert scale between “strongly disagree” (1) and “strongly agree” (5). Higher scores designate higher degrees of alexithymia with a possible score ranging between 20 and 100. Cut-off scores have been developed with scores ≤50 suggesting no alexithymia, scores between 51 and 60 indicating borderline alexithymia, and scores ≥61 suggesting alexithymia [50]. The TAS-20 has been demonstrated to be a valid and reliable measure of alexithymia [40] with an internal consistency of α=.81 and test-retest reliability of .77 (*P*<.01).

#### Bermond–Vorst Alexithymia Questionnaire

The Bermond–Vorst Alexithymia Questionnaire (BVAQ) [11] is also a measure of alexithymia that aims to assess the alexithymia construct more comprehensively across 2 subdimensions, namely the cognitive and the affective. Measuring these 2 subdimensions aids in distinguishing between participants with type 1 and type 2 alexithymia. In total, there are 40 items rated on a 5-point Likert scale, with higher scores indicating higher levels of alexithymia. This model of alexithymia and the BVAQ have demonstrated strong validity and reliability [10,11].

#### DASS-21

The DASS-21 [46] is a self-report questionnaire that is designed to measure depression, anxiety, and stress/tension levels. Each of the 3 subscales contains 14 items that are scored on a 4-point Likert style scale, which ranges from 0, “did not apply to me at all,” to 3, “applied to me very much, or most of the time.” Total scores are calculated by summing the scores from the 3 subscales, with higher scores indicating more of that construct. The DASS has good convergent and divergent validity, with strong internal consistency and reliability, with Cronbach alphas observed at .94, .87, and .91 for depression, anxiety, and stress, respectively [51]. Additionally, the DASS has established normative data for adults with an ASD [52].

#### AMSE

The AMSE 2.2 [43] is an 8-item clinician observational assessment that aims to structure the documentation and observation of social, communicative, and behavioral function in people with ASD. The AMSE takes place during a clinical interview without adding extra work or time during the examination. Scores higher than 4 predict ADOS [53] classification with a sensitivity of 94% and specificity of 81% [44]. Interrater agreement is high at 0.97, and internal consistency was fair with a Cronbach α of .72 [43].

#### WASI-ll

The WASI-II [47] is an individual, clinician-administered measure of cognitive intelligence based on the Wechsler Adult Intelligence Scale - Fourth Edition (WAIS-IV) [54]. The WASI-II includes 4 subtests: block design, vocabulary, matrix reasoning, and similarities. It produces 3 metrics: Full-Scale IQ, Verbal IQ, Performance IQ. The WASI-II correlates highly with the WAIS-IV and has good reliability and validity [55].

#### AQ-10

The AQ-10 [42] is a short-form, 10-item, self-assessment instrument for measuring autistic traits based on the AQ-10 [56]. Each of the 10 items contains a statement such as “I find it easy to 'read between the lines' when someone is talking to me.” Responses are made on a 4-point scale ranging from “Definitely Agree” to “Definitely Disagree.” One point is scored if the respondent selects “Definitely Agree” or “Slightly Agree” on each of items 1, 7, 8, and 10. One point is scored for “Definitely Disagree” or “Slightly Disagree” on each of items 2, 3, 4, 5, 6, and 9*.* The AQ-10 has good construct validity and adequate internal consistency with a Cronbach alpha of .85 [42].

#### Questionnaire of Task Satisfaction

In addition to these measures, a self-developed questionnaire of satisfaction, acceptance, and enjoyment of the task will also be designed to assess how viable this method of training is for people with alexithymia and ASD (Multimedia Appendix 2). It will include 5 statements about the study task, with responses on a 5-point scale. Responses will range from 1 (Not very true) to 5 (Very true). Higher scores for each question indicate that the statement applied more strongly to the individual. Examples of the items are “The task made me uncomfortable” and “I feel like this task helped me.”

### Equipment

**Figure 1:**
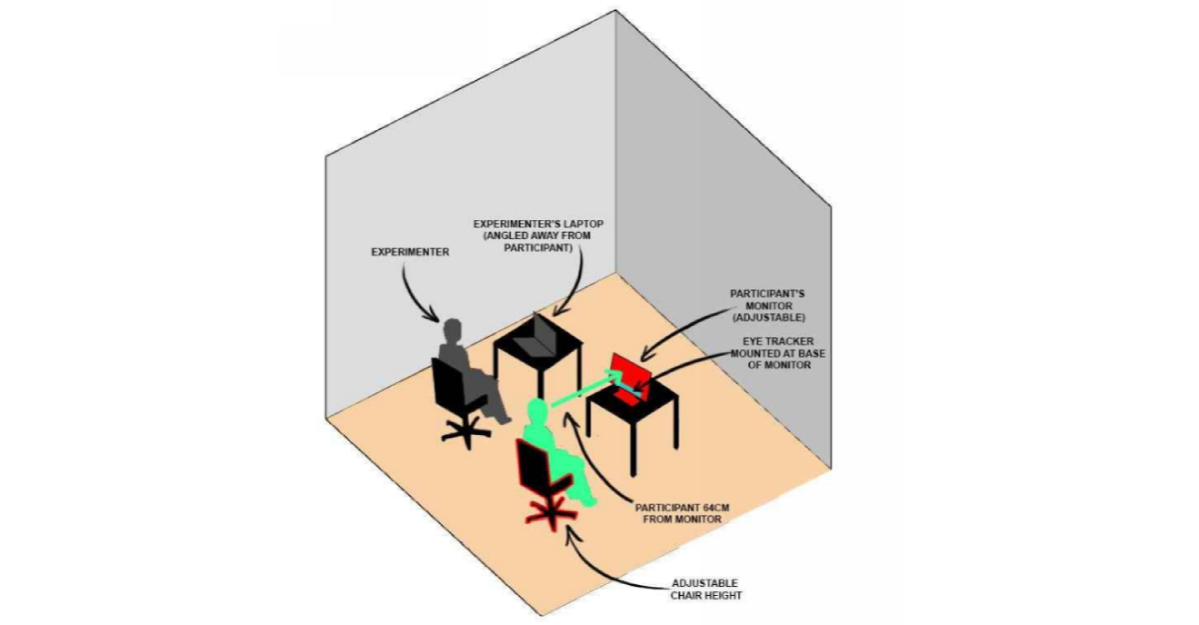
Physical layout of experiment and eye-tracker.

The Tobii Pro x3-120 Eye Tracker (TPX3) is a screen-based eye tracker that tracks the gaze point on a screen. The TPX3 has been certified to meet European standard EN 62471, which indicates that it is not harmful to the human eye. The TPX3 has a sampling rate of 120 Hz and a large head movement box (area where the user’s head can move and still be tracked). Only the gaze point 2D coordinates on the screen for each eye will be used. The TPX3 will attach to the bottom of a computer monitor that is ~64 cm in front of the participant. Both the chair and the participant’s screen can adjust for height to make sure the participant’s head is within the head movement box as indicated in Figure 1. The chair and screen height will be adjusted based on the setup procedure within the iMotions software prior to running the iMotions software eye-tracker calibration process.

### Primary Outcome

The primary outcome being assessed is an increase in FEER. This will be measured by comparing the percent of correctly identified facial emotion expressions from the baseline assessment task to the instructed mimicry experimental task. Additionally, the percent of correctly identified expressions will also be compared between the control condition and the intervention condition. This will provide 2 primary metrics: before-after percent improvement and control group to experimental group percent improvement.

### Intervention

The task will be delivered in-person at the Federation University Psychology Clinic in Ballarat, Australia, by the primary author in a small counselling room. The room will have 2 computers on a desk, each with separate monitors and peripherals (one for the participant and the other for recording eye-tracking data). Participants will take part in the task 1 participant at a time based on a mutually agreed upon time and date. Participants will only undergo this task once, and it is expected to take approximately 30 minutes based on prior testing where a small neurotypical group took approximately 25 minutes to complete the task in a usability test. The intervention is a software-based FEER training task that presents a series of different facial expressions and requires individuals to firstly mimic and then select which emotional expression they saw. The software runs on Microsoft Windows 10 and requires only minimal system requirements and a mouse.

The intervention task condition will begin with the software presenting a basic description of 6 facial expressions (fear, sadness, happiness, anger, disgust, surprise) and tips on how to spot each expression. Next, participants will be verbally advised by the experimenter and by on-screen text that a series of emotion recognition trials will begin shortly. Further, they will be told that after each emotion is displayed that they should try their best to mimic (copy) the observed facial expression with their own face. Once they have mimicked the expression, they should then use the 6 labelled buttons on the right-hand side (or associated keyboard shortcut) to choose which emotion was displayed. After selecting the answer using 1 of the 6 buttons, a 5-point Likert scale on the computer screen will ask the participant “How well did you mimic the expression?” with responses ranging from 1 “not very well” to 5 “very well.” This will repeat for each of the 36 trials. The trials will include various models of both men and women depicting the 6 emotions.

**Figure 2:**
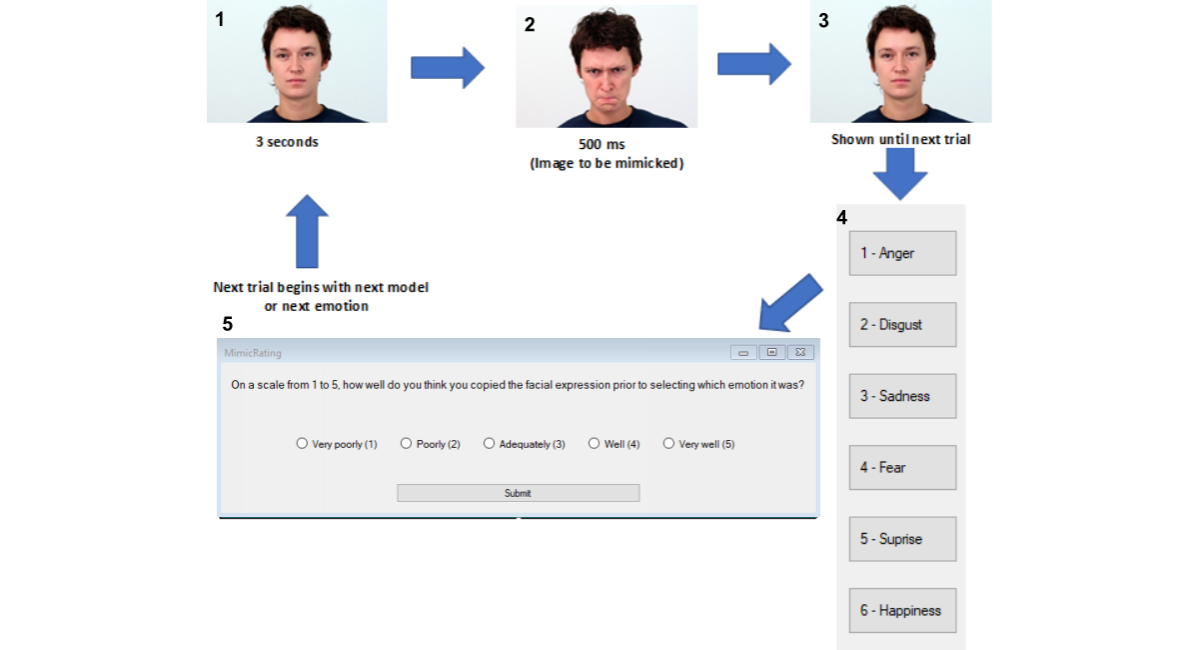
Task sequence and stimulus example.

The sequence of each trial in the task is depicted in Figure 2 with accompanying numbered indexes. Each trial will begin with a neutral expression image displayed on the screen (index 1). After this image has been displayed for 3 seconds, it will quickly swap to another image from the same model, except this image will depict an emotional expression (eg, angry or sad, index 2). After 500 milliseconds, this image will be replaced with the original neutral image (index 3), and the 6 buttons with emotion labels will appear on the right-hand side (index 4). Once the participant selects the emotion using the 6 buttons, the trial will conclude with the 5-point rating of how well the participant felt they copied the expression (index 5).

In a task that aims to test or train FEER ability, it is important to ensure that the emotional stimuli tested against are true exemplars of the emotion they purport to signal. It is also important that the expressive images in the baseline test and the experimental tasks are similar in recognition difficulty. This will reduce the risk of improvements being attributed to easier expressions in the experimental tasks compared to the baseline task. This task used the Warsaw Set of Emotional Facial Expression Pictures (WSEFEP) [57]. See Multimedia Appendix 3 for a description of these pictures and how pictures were selected for each condition. See Figure 2 for an example of the pictures.

### Comparator/Control Group

This study includes both a before-after, within-group comparator and a between-groups control group. To make the before-after comparison, participants will first partake in a baseline assessment task of emotion recognition before randomization to one of the experimental conditions. This baseline assessment task will be the same as the intervention task; however, participants will not be instructed to mimic the facial expressions prior to selecting an expression.

In addition to the before-after comparator, there will be a control condition. The control condition will also undergo the baseline assessment task; however, instead of participating in the mimicry intervention task, they will undergo a similar task with no mimicry element. This task will be identical to the instructed mimicry intervention task; however, instead of being instructed to mimic the facial expression, they will be instructed to “pay particular attention to the entire face prior to selecting an expression.” This instruction is aimed to match for attention levels.

**Figure 3:**
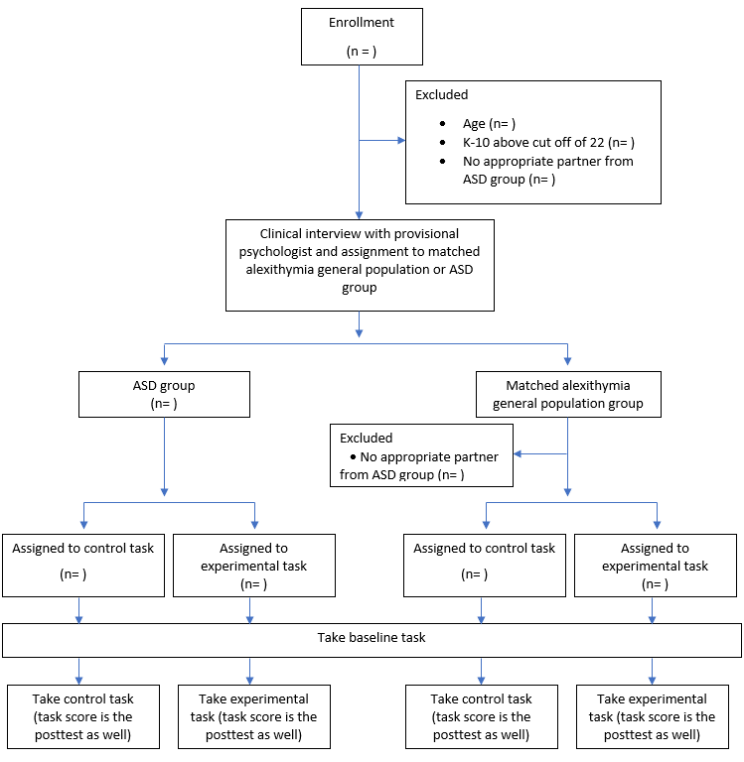
Participant flow diagram. ASD: autism spectrum disorder; K-10: Kessler Psychological Distress Scale.

A final point of comparison will be that participants will be first broken up into 2 groups, namely the AM group and ASD group. This comparison will provide the ability to analyze if any intervention effects only occur or are stronger in people with ASD, alexithymia, or the general population. The participant flow diagram can be seen in Figure 3.

### Randomization

Participants will be randomized using permuted block randomization with stratification across age and gender to receive either the control or experimental task condition with equal numbers from both the ASD group and AM group receiving each task.

### Sample Size Calculation

Based on the effect sizes of 2 previous studies measuring similar constructs and similar tasks [32,35], to achieve a medium to large effect (eg, Gpower f^2^ test = 0.27) with a significance of 5% (*P*=.05) and power of 80%, a sample size of 58 will be required to demonstrate statistical significance for the primary hierarchal regression and analysis of variance (ANOVA). Given an expected attrition of 10%, a total of 64 participants will be recruited.

### Statistical Analyses

The data will be analyzed using SPSS, Version 25. Alexithymia will be analyzed to aid in matching the AM group and ASD group on alexithymia. To ensure that the ASD and AM groups, and also the experimental and control groups, are comparable, means and standard deviations of participant characteristics will also be compared with tests of difference.

The study design is a mixed factor, 2x2x2, within-between design (see Table 2), and therefore, the most appropriate test of difference is a mixed factorial ANOVA, set up according to Table 2 with IQ, depression, and anxiety variables added as covariates. This will provide analyses of whether the instructed mimicry intervention task was effective by comparing baseline scores to posttest scores. Additionally, it will provide the ability to compare posttest scores between the control group and the intervention group. Finally, this analysis will have the additional ability to discern these differences based on group (ASD group and AM group).

In addition to the mixed factorial ANOVA, a hierarchical regression analysis will also be conducted (see Table 3) to determine the predictive ability of alexithymia on the primary outcome variable.

**Table 2:** Experimental group and intervention design.

Time point	ASD^a^ group (n=~32)		AM^b^ group (n=~32)	
	Control task condition (n=~16)	Mimicry task condition (n=~16)	Control task condition (n=~16)	Mimicry task condition (n=~16)
Pretest	FEER^c^ task score	FEER task score	FEER task score	FEER task score
Posttest (the intervention task is the posttest)	FEER task score	FEER task score	FEER task score	FEER task score

^a^ASD: autism spectrum disorder.

^b^AM: alexithymia-matched general population.

^c^FEER: facial emotion expression recognition.

**Table 3:** Hierarchal regression stages for analysis.

Stage	Variables
Stage 1	IQ, age, gender
Stage 2	+Autism
Stage 3	+Alexithymia
Stage 4	+Intervention

### Hypotheses

It is hypothesized that the condition that receives the mimicry task will show significantly better emotion recognition improvement scores (posttest minus baseline); the degree of alexithymia will negatively predict baseline scores; the degree of alexithymia will predict improvement scores (posttest minus baseline); when matched on alexithymia, the general population and ASD population will not significantly differ in emotion recognition improvement scores (posttest minus baseline); and when matched on alexithymia, the AM group and ASD group will not significantly differ on baseline emotion recognition scores.

## Results

### Ethics Approval and Trial Registration

This study will meet the ethical guidelines outlined in the National Statement on Ethical Conduct in Human Research (NHMRC, ARC & UA) [58]. This study has been granted full approval from the Federation University Australia Human Research Ethics Committee (Project # A19-036) and is registered with the Australian New Zealand Clinical Trial Registry (ACTRN: ACTRN12619000705189).

### Timeline

As of June 2021, the project has not begun recruitment. The project is predicted to begin participant recruitment in the third quarter of 2021, with data collection beginning as eligible consenting participants make contact.

## Discussion

This paper describes, to our knowledge, the first study to assess an instructed mimicry task to increase emotion recognition ability in people with alexithymia and ASD. Despite the formulation of the alexithymia hypothesis, very few studies have assessed the impact of alexithymia in interventions aimed at increasing social functioning and in particular, emotion recognition. Additionally, to our knowledge, no other study has explored the benefits of instructed mimicry on emotion recognition in people with ASD and alexithymia. This mixed factor, 2x2x2, within-between design will assess the efficacy of the instructed mimicry task to increase FEER. This study design will enable the assessment of baseline and improvement scores individually for people with and without ASD while controlling for levels of alexithymia. This will allow analyses of how this intervention uniquely benefits ASD without the interference of alexithymia, which is known to frequently co-occur in ASD [9]. If this task is successful at increasing emotion recognition, it could provide easily accessible training to those with ASD, alexithymia, and other disorders that typically have high rates of alexithymia such as schizophrenia [59].

One proposed benefit of this intervention for people with alexithymia and ASD is that the software does not require supervision. This will likely benefit these populations as they typically enjoy computer-based interaction rather than social interaction, as indicated by previous studies (eg, [60]). This has the added benefit of improving access to the task as it could be used on a home computer, which may eliminate a key barrier to treatment.

Despite the benefits of this task and study design, some limitations are present. First, matching the ASD and AM groups on levels of alexithymia improves our ability to exclude alexithymia as a contributing factor of emotion recognition accuracy. Unfortunately, it also means that other confounding factors may have been systematically introduced when matching on alexithymia. This limitation will make it difficult to attribute any benefits of the intervention task specifically to autism. Additionally, when comparing emotion recognition accuracy, it will limit our ability to suggest whether emotion recognition difficulties are related to ASD or to alexithymia, as per the alexithymia hypothesis.

A second limitation is the once-off nature of this intervention. While similar tasks have shown improvement over one session (eg, [61]), it would be ideal to administer the intervention several times over a longer time period and measure in between. Given that the primary variable, emotion recognition, is measured during the task (the intervention task is the posttest), this could be done by having participants complete the task at home for a specific amount of time and intervals.

In summary, it has been suggested that emotion recognition problems in ASD are instead due to co-occurring alexithymia. This study matches an ASD and non-ASD general population group (the AM group) on alexithymia to improve the ability to detect if this is true. Further, this study design will aid in detecting if the instructed mimicry intervention benefits people specifically with ASD or if it more broadly aids people with alexithymia. If successful, this adjunct intervention will be cost-effective and easy to implement while also removing entry barriers as it could be used at home on a personal computer.

## Appendix

Multimedia Appendix 1 Workflow of participants through in-person segments of study.

Multimedia Appendix 2 Questionnaire of Task Satisfaction.

Multimedia Appendix 3 Selection process of pictures to match task difficulty between the baseline task and the experimental task.

## Abbreviations

ADOS: Autism Diagnostic Observation Schedule
AM: alexithymia-matched general population
AMSE: Autism Mental Status Exam
ANOVA: analysis of variance
AQ-10: Autism Spectrum Quotient
ASD: autism spectrum disorder
DASS-21: Depression Anxiety Stress Scales-21
FEER: facial emotion expression recognition
K-10: Kessler Psychological Distress Scale
PCF: predictive coding framework
PP: provisional psychologist
TAS: Toronto Alexithymia Scale
WAIS-IV: Wechsler Adult Intelligence Scale - Fourth Edition
WASI-II: Wechsler Abbreviated Scale of Intelligence
WSEFEP: Warsaw Set of Emotional Facial Expression Pictures
