# Supplementary material for: The Impact of a Novel Mimicry Task for Increasing Emotion Recognition in Adults with Autism Spectrum Disorder and Alexithymia: Protocol for a Randomized Controlled Trial
Source: JMIR Res Protoc. 2021 Jun 17;10(6):e24543. doi: 10.2196/24543 (PMC8386358; doi:10.2196/24543)
Supplement: Multimedia Appendix 1 [file resprot_v10i6e24543_app1.docx]

**Appendix 1: Workflow of participants through in-person segments of study**

**Stage 2. Clinical interview (40 minutes)**

1. Arrival at clinic
2. Provisional psychologist provides information of the upcoming assessment and confirms informed consent.
3. Based on response from pre-screening questions, the provisional psychologist will ask the participant about their diagnosis of ASD, how it was made and whether they consent to past diagnostic report(s) being viewed.
4. Provisional psychologist will then administer the WASI-II and the DASS 21.
5. additionally, throughout these assessments the provisional psychologist will observe the participant so that they may complete the AMSE based on their interaction with the participant.

**Stage 3. Experiment (40 minutes)**

1. Participants choose to take a break or proceed directly to another room of the clinic to begin the experiment (stage 3).
2. Participants asked to complete the BVAQ
3. Participants randomised with stratification across age and gender to the control or experimental task
4. Participants will be seated at a computer and set 64cm from the monitor.
5. Galvanic Skin Conductance equipment setup and tested with Imotions^©^ software for signal.
6. Participant advised on the Eye-tracking calibration procedure.
7. Eye-tracker calibrated using Imotions^©^ software which has the user direct their gaze towards dots on the computer monitor.
   1. If calibration fails or is of a low quality, sitting position will be re-assessed, instructions given to participant again, followed by repeating the calibration process.
8. Participants will be briefed on the baseline assessment task.
9. Participants will undergo the baseline task.
10. Participants will be briefed on the intervention task.
    1. If assigned to the control condition task, participant will be instructed to “pay particular attention to the entire face prior to selecting an expression”
    2. If assigned to the mimicry condition task, participant will be instructed “that after each emotion is displayed, they should try their best to mimic the facial expression that they saw with their own face before selecting an expression.”
11. Participants will undergo the experimental task
12. The Galvanic Skin Conductance equipment will be removed, and the participant taken to another desk to complete the Questionnaire of Task Satisfaction.
13. The study will conclude with a debriefing session where the participant will be allowed to ask the experimenter questions about the study and task.

**Stage 1. Online screening (15 minutes)**
