# Supplementary material for: The Impact of a Novel Mimicry Task for Increasing Emotion Recognition in Adults with Autism Spectrum Disorder and Alexithymia: Protocol for a Randomized Controlled Trial
Source: JMIR Res Protoc. 2021 Jun 17;10(6):e24543. doi: 10.2196/24543 (PMC8386358; doi:10.2196/24543)
Supplement: Multimedia Appendix 2 [file resprot_v10i6e24543_app2.docx]

**Appendix 2: Questionnaire of Task Satisfaction**

Participant ID: ___________ Date: ____________

On a scale from 1 to 5, how true are the following statements about the task you completed in this study? Please circle one number.

1: Not very true 5: Very true

1. I found the task stressful: 1 2 3 4 5
2. The task made me uncomfortable: 1 2 3 4 5
3. I enjoyed the task: 1 2 3 4 5
4. I would use this task as a training method: 1 2 3 4 5
5. I feel like this task helped me: 1 2 3 4 5

**(Optional) Any other comments you would like to add:**
