# Supplementary material for: The Impact of a Novel Mimicry Task for Increasing Emotion Recognition in Adults with Autism Spectrum Disorder and Alexithymia: Protocol for a Randomized Controlled Trial
Source: JMIR Res Protoc. 2021 Jun 17;10(6):e24543. doi: 10.2196/24543 (PMC8386358; doi:10.2196/24543)
Supplement: Multimedia Appendix 3 [file resprot_v10i6e24543_app3.docx]

**Appendix 3: Selection process of pictures to match task difficulty between the baseline task and the experimental task**

The Warsaw Set of Emotional Facial Expression Pictures were developed using a method that aims to have the models present an expression from a genuine and felt emotion. Further, the image set has been validated using both untrained judges, and judges trained in the Facial Action Coding System [FACS; 1]. This provides additional verification that the images are true exemplars of one of the six basic and universal emotions. In addition to this verification, three metrics of the images are provided which includes the percent agreement the judges agreed on which emotion was being presented, the purity of the emotion (i.e. to what degree is this expression pure, versus containing elements of other emotional expressions), and finally, the percent intensity of the emotion. The WSEFEP includes 30 models, each presenting a neutral (non-emotional) expression, and each of the six basic emotions to total 210 images. For this task, and the baseline test, each of the six basic emotions is presented six times, three times by a male model and three times by a female model to total 36 unique emotional displays.

To select the subset of images to be used out of the 210 WSEFEP images, any emotional image was removed if its agreement percentage was below 70% or if its purity rating was below 0.7. One exception to this was for the fear emotion, a purity rating of 0.6 or above was accepted to get an adequate number of fear images. It is documented that the muscles used for the fear expression overlap heavily with surprise [1], which is likely the cause of the low purity metric for the fear images. Images were then selected based on which model had the least images excluded based on the above metrics. Where a model had excluded images, they were replaced using the same emotion from a model of the same gender.

**Appendix 3: Continued**

From this point, the resulting images included 72 emotionally expressive pictures, each with their related models' neutral image. These remaining images were separated into two groups, with one being for the baseline task, and the other for the experimental task. During this process, care was taken to have similar levels of the three metrics (agreement percent, purity and intensity percent) in each group, while also having an equal number of males, and females in each group and each emotion equally represented. To check that both groups of images were similar, an independent samples t-test was conducted between the groups on the three metrics. The results suggested that there was no statistically significant difference between the groups on agreement percent, purity or intensity as observed in table 4 and table 5. This suggests that the difficulty to recognise the images in the baseline test, and the experimental tasks will be similar.

|  | **Condition** | ***n*** | ***SD*** | **Mean** |
| --- | --- | --- | --- | --- |
| Agreement percentage | Baseline | 36 | 7.041 | 88.833 |
|  | Experimental | 36 | 7.880 | 89.750 |
| Expression purity | Baseline | 36 | .064 | .765 |
|  | Experimental | 36 | .090 | .775 |
| Expression intensity | Baseline | 36 | .073 | .710 |
|  | Experimental | 36 | .061 | .723 |

*Table 4. Independent samples t-test for metrics of emotion expression images presented during the two conditions*

|  | ***t*** | ***df*** | ***p*** | **Mean difference** | **95% Confidence Interval of the Difference** | |
| --- | --- | --- | --- | --- | --- | --- |
|  |  |  |  |  | **Lower** | **Upper** |
| Agreement percentage | -.521 | 70 | .604 | -.917 | -4.429 | 2.600 |
| Expression purity | -.558 | 63.427 | .579 | -.010 | -.0470 | .027 |
| Expression intensity | -.819 | 70 | .415 | .013 | -.045 | .019 |

**Appendix 3: Continued**

Table 5. Independent samples t-test for metrics of emotion expression images presented during the two conditions

References

1. Ekman, P., W.V. Friesen, and J.C. Hager, *Facial action coding system: the manual*. 2002, Salt Lake City, Utah: Research Nexus. 512.
